# Dosimetric rationale and preliminary experience in proton plus carbon-ion radiotherapy for esophageal carcinoma: a retrospective analysis

**DOI:** 10.1186/s13014-023-02371-9

**Published:** 2023-12-01

**Authors:** Ningyi Ma, Xue Ming, Jian Chen, Kai-Liang Wu, Jiade Lu, Guoliang Jiang, Jingfang Mao

**Affiliations:** 1 Department of Radiation Oncology, Shanghai Proton and Heavy Ion Center, Fudan University Cancer Hospital, Shanghai Key Laboratory of radiation oncology (20dz2261000), Shanghai Engineering Research Center of Proton and Heavy Ion Radiation Therapy, 4365 Kang Xin Road, Shanghai, 201315 China; 2 Department of Medical Physics, Shanghai Proton and Heavy Ion Center, Shanghai Key Laboratory of radiation oncology (20dz2261000), Shanghai Engineering Research Center of Proton and Heavy Ion Radiation Therapy, Shanghai, China

**Keywords:** Esophageal carcinoma, Proton radiotherapy, Carbon ion radiotherapy, Toxicity, Survival, Pencil-beam scanning

## Abstract

**Background:**

Concurrent chemoradiotherapy has been standard of care for unresectable esophageal carcinoma. There were no reports on proton radiotherapy (PRT) plus carbon-ion radiotherapy (CIRT) with pencil beam scanning (PBS) for esophageal carcinoma. This study evaluated the tolerability and efficiency of proton and sequential carbon-ion boost radiotherapy for esophageal carcinoma.

**Methods:**

From April 2017 to July 2020, 20 patients with primary esophageal carcinoma at stages II–IV were treated with PRT plus sequential CIRT with PBS. A median relative biological effectiveness-weighted PRT dose of 50 Gy in 25 fractions, and a sequential CIRT dose of 21 Gy in 7 fractions were delivered. Respiratory motion management was used if the tumor moved > 5 mm during the breathing cycle. A dosimetric comparison of photon intensity-modulated radiotherapy (IMRT), PRT, and CIRT was performed. The median times and rates of survivals were estimated using the Kaplan–Meier method. Comparison of the dose-volume parameters of the organs at risk employed the Wilcoxon matched-pairs test.

**Results:**

Twenty patients (15 men and 5 women, median age 70 years) were included in the analysis. With a median follow-up period of 25.0 months, the 2-year overall survival and progression-free survival rates were 69.2% and 57.4%, respectively. The patients tolerated radiotherapy and chemotherapy well. Grades 1, 2, 3, and 4 acute hematological toxicities were detected in 25%, 30%, 10%, and 30% of patients, respectively. Grades 3–5 acute non-hematological toxicities were not observed. Late toxicity events included grades 1, 2, and 3 in 50%, 20%, and 10% (pulmonary and esophageal toxicity in each) of patients. Grades 4–5 late toxicities were not noted. PRT or CIRT produced lower doses to organs at risk than did photon IMRT, especially the maximum dose delivered to the spinal cord and the mean doses delivered to the lungs and heart.

**Conclusions:**

PRT plus CIRT with PBS appears to be a safe and effective treatment for esophageal carcinoma. PRT and CIRT delivered lower doses to organs at risk than did photon IMRT. Further investigation is warranted.

## Background

Esophageal carcinoma is the seventh most common malignancy and the sixth leading cause of cancer-related deaths worldwide [1]. In China, it ranks the sixth and fourth, respectively [2]. In contrast to adenocarcinoma, which is prevalent in European and American developed countries, squamous-cell carcinoma (SCC) is the leading (> 90%) type of malignant esophageal tumor in China. Compared to adenocarcinoma, SCC is more aggressive and exhibits more extensive lymph node metastasis and local infiltration and a worse prognosis [3, 4].

Recently, definitive concurrent chemoradiotherapy with a radiotherapy dose of 50–50.4 Gy has been considered the standard treatment for locally advanced esophageal carcinoma that is unresectable due to tumor position or invasion of surrounding tissues/organs and for those who cannot tolerate or refuse surgery [5]. An increased dose (e.g., more than 60 Gy) did not confer superior survival in esophageal carcinoma in clinical studies, [6] and toxicities induced by treatment might be a partial explanation. Technical improvements in highly conformal therapy translate into an increased radiation dose without increased treatment-related toxicities, which was expected to improve clinical outcomes in these patients.

A new type of treatment, particle (proton and carbon ion) radiotherapy (RT), presents advantages in physics over photon therapies. Multiple studies comparing dosimetric distributions have demonstrated that in esophageal carcinoma, particle beams can deliver a higher dose to the tumor while sparing the healthy organs at risk (OARs) such as the heart and lungs, thereby reducing treatment-related cardiopulmonary toxicity [7–9]. A retrospective study of proton radiotherapy (PRT) found improved clinical survival compared to photon RT and similar treatment-related toxicities in locally advanced esophageal carcinoma [10]. Moreover, a prospective trial showed that PRT and photon RT produced similar clinical survival; however, a reduced risk and severity of adverse effects were observed in the former [11].

Additionally, carbon ions produce more severe biological damage than do protons or photons owing to their higher linear energy transfer (LET) and could theoretically be more effective for radio-resistant tumors [12]. In the last 30 years, carbon-ion radiotherapy (CIRT) has produced significant clinical results in bone and soft-tissue sarcomas, head and neck cancers, hepatocellular carcinoma, prostate cancer, locally advanced cervical squamous-cell carcinoma, recurrent rectal cancer, and lung cancer [13–22]. Although no reports of definitive CIRT in locally advanced esophageal carcinoma have appeared, this treatment has been recommended for neoadjuvant radiotherapy in Japan [23]. Concerns of irreversible damage to the esophagus from neutron radiotherapy with high linear energy transfer in definitive radiotherapy or from CIRT alone should be considered cautiously [24]. Clinical practice using PRT combined with sequential CIRT for esophageal carcinoma was available at our center. Theoretically, PRT can eliminate radiosensitive tumor cells and mostly spare the OARs, and a sequential CIRT boost can effectively eliminate the remaining radio-resistant cells, which might provide satisfactory outcomes. We find no reports of PRT plus CIRT using pencil beam scanning (PBS) technology for esophageal carcinoma.

Therefore, this retrospective study aimed to investigate the safety and efficiency of PRT plus sequential CIRT boost in esophageal carcinoma and to perform a dosimetric comparison with photon intensity-modulated radiotherapy (IMRT).

## Methods

### Patient inclusion criteria

Patients who met the following criteria were included in this retrospective study: (1) pathological diagnosis of SCC, adenocarcinoma, or adenosquamous carcinoma; (2) stages I-III and IV (limited to patients with metastasis only to paraesophageal supraclavicular and cervical non-regional lymph node); (3) no esophageal surgery or thoracic irradiation prior to particle radiotherapy; and (4) received PRT combined with sequential CIRT boost therapy. The patients who received photon plus particle RT or a diagnosis of failure after surgery or RT were excluded from the study.

#### Treatment regimen

A total of four to six cycles of chemotherapy with fluorouracil- or platinum-based regimens were recommended during the whole treatment schedule. Highly recommended were thoracic PRT and sequential CIRT boost therapy with concurrent chemotherapy consisting of 5-fluorouracil (5-Fu) at 1800 mg/m^2^ continuous intravenous 72 hours on day 1; plus cisplatin (DDP) at 25 mg/m^2^, days 1–3; every 4 weeks; or paclitaxel at 135 mg/m^2^ or docetaxel at 75 mg/m^2^, day 1; plus cisplatin at 25 mg/m^2^, days 1–3; every 4 weeks.

### Clinical practice of particle radiotherapy

Patients were set up routinely with a head-neck-shoulder or a trunk immobilization system according to the location of the esophageal carcinoma. The movement of tumor lesions and surrounding organs under free breathing was evaluated by fluoroscopy prior to simulation computed tomography (CT) scanning. If the motion of the tumor and/or surrounding OARs within the range of the particle trajectories was > 5 mm, respiratory gating or active breathing control was applied; otherwise, free breathing was used for simulation and particle delivery.

Gross tumor volume (GTV) was confirmed by CT, X-ray barium-meal imaging, positron emission tomography/CT (PET/CT), and gastroscopy. iGTV (internal gross tumor volume) was formed by combining GTVs across all 10 phases of respiratory gating. The high- or low-risk clinical target volume (CTV__highrisk_ or CTV__lowrisk_) typically included 1 or 3 cm of the esophagus craniocaudally adjacent to the GTV/iGTV and a uniform 0.5–0.6 cm margin in all directions. The involved lymph nodes with a uniform expansion of 0.5-0.6cm was defined as CTV__highrisk_. Prophylactic lymph nodal irradiation was not applied except that supraclavicular and superior mediastinal lymph node regions were irradiated as CTV__lowrisk_ for cervical esophageal carcinoma. The planning target volume (PTV) was generated by considering the range uncertainties and set-up errors. On average, the CTV was expanded 6 mm laterally, 6-8 mm at the entrance, and 8–12 mm in the distal direction of the beam view to form the PTV.

Radiation doses were prescribed as relative biological effectiveness (RBE)-weighted dose (D_RBE_) expressed in units of Gray (Gy) for both proton and carbon ions. The prescribed dose of PRT was delivered to CTV__lowrisk_ and CTV__highrisk_, then the CIRT boost was delivered to CTV__highrisk_ only. The requirement for dose coverage was as follows: at least 99% of GTV/iGTV was covered by 95% of the prescription dose, 99% of the CTV was covered by 95% of the prescription dose to the CTV, and 90% of the PTV was covered by 90% of the prescription dose unless this would have exceeded the dose constraints.

The dose constraints for the OARs stipulated that the maximum dose (D_max_) of the esophagus and trachea should be less than 107% of the prescription doses. The D_max_ to the spinal cord was also required to be < 45 Gy. The mean doses to both lungs were required to be < 14 Gy. If the doses to OARs could not meet these requirements, the dose coverage to the radiation targets was acceptably compromised.

The particle (carbon ion or proton) RT plans were designed using a commercial treatment planning system, Syngo PT Planning (Siemens Healthcare Systems, Erlangen, Germany), and intensity modulated PRT or CIRT (IMPT or IMCT) was realized with single beam or multiple-beam optimization strategy using pencil-beam techniques. The local effective model (LEM) was utilized to calculate the biological dose for carbon ion and 1.1 was set as the relative biological effectiveness for proton [25]. A fixed oblique gantry (45°) was applied for multiple-field irradiation, and usually two to three beams were applied via couch rotation. The full width at half maximum (FWHM) of 10.0 mm and the grid size of 2.0 mm were applied in the lateral beam spot density in both proton and carbon-ion plans. A range shifter, with 2 cm of thickness, was used for the shallow target in both proton and carbon-ion plans. Ripple filter of 3 mm-thickness that broadens the Bragg peak served as the energy modulator in the carbon-ion plans. Both proton and carbon-ion plans were calculated with 3 mm in grid. During particle therapy, CT review and dose distribution recalculation were performed weekly. If poor dose coverage or overdoses to OARs were evident, treatment replanning was required.

### Dosimetric comparisons among IMRT, IMPT, and IMCT

Comparisons among plans were conducted for all enrolled patients. The same CTV__lowrisk_ and GTV/iGTV were used for the IMRT, IMPT, and IMCT plans, while the PTVs for IMRT were generated by adding a uniform margin of 6 mm to the CTV. A simulated IMRT plan was generated for comparison of dosimetric parameters, which was designed using Eclipse (Varian, Palo Alto, California, USA). Five to seven beams were occupied in the photon plans with a calculation grid size of 2.5 mm. For dosimetric comparison, the prescription D_RBE_ of 69.3 Gy in 21 fractions was used for IMCT and IMPT plans. The same fractionation using absorbed dose was prescribed in IMRT plans and a similar target dose coverage was pursued. The OAR dose constraints used in the dosimetric study were the same for IMRT, IMPT, and IMCT as in clinical practice, which was described above for particle RT, except that the Dmax to the spinal cord was required to be < 50 Gy in IMRT plans.

The following parameters were extracted from the dose-volume histograms (DVHs): D_max_ to the main bronchial tree (MBT, including the trachea, the carina, and the right and left main bronchi as far as the openings of the segmental bronchi) and spinal cord; the dose that 1% of the volume received (D_1%_) of the MBT and spinal cord; the percentage volume of the lungs and heart that received 5–60 Gy [V5–V60]; and the mean doses (D_mean_) to each.

### Follow-up and statistical analyses

Follow-up was conducted weekly during radiotherapy, every 3–4 months in the first and second year after radiotherapy, every 6 months in the third and fourth year, and every year thereafter. During radiotherapy, a physical examination and a complete blood count were conducted once a week (twice a week if concurrent chemotherapy was administered), X-ray barium-meal imaging was performed every 2 weeks, and hepatic/renal function was tested prior to concurrent chemotherapy. Physical examination, complete blood count, hepatic/renal function, X-ray barium-meal imaging, and CT were performed every post-RT medical visit. Gastrointestinal endoscopy was performed if clinical failure or any severe gastrointestinal side effect was suspected detected by physical examination, X-ray barium-meal imaging, CT, etc.

The following data were collected: (1) treatment toxicities quantified by the Common Terminology Criteria for Adverse Events version 4.0 (CTCAE); (2) treatment response as measured by CT scanning and X-ray barium-meal imaging, and any further PET/CT scanning and/or endoscopic or aspiration biopsy if needed; and (3) the rates and median times of overall survival (OS), progression-free survival (PFS), locoregional progression-free survival (LRPFS), and distant metastasis-free survival (DMFS). Observation of all events started from the initiation of radiotherapy until an event occurred or until the last follow-up, whichever came first.

The rates and median times of OS, PFS, LRPFS, and DMFS were estimated by the Kaplan–Meier method. The treatment planning DVH parameters were compared by the Wilcoxon matched-pairs test. The STATA statistical software package was used for statistical analyses (version 11.0; StataCorp LP, Texas, USA). A p-value < 0.05 was considered statistically significant.

## Results

### Patient characteristics

From April 2017 to July 2020, 21 consecutive patients who met the inclusion criteria received particle radiotherapy at our institution. One patient was lost to follow-up. Finally, 20 patients were included in this study. The characteristics of the study sample are summarized in Table 1.

**Table 1:** Characteristics and treatment details of patients with esophageal carcinoma included in this study (N = 20)

Characteristics	Data
Sex, n (%)	
Male/ Female	15 (75) / 5 (25)
Age, years, median (range)	70 (54–85)
Tumor location, n (%)	
Cervical/ Upper/ Middle/ Lower thoracic	2 (10) / 8 (40) / 6 (30) / 4 (20)
SUV_max_	
mean	12.1
median, range	10.7 (3.6–31.6)
T classification, n (%)	
T2/ T3/ T4	4 (20) / 15 (75) / 1 (5)
N classification, n (%)	
N0 / N1/ N2	5 (25) / 10 (50) / 5 (25)
Stage, n (%)	
II/ III/ IV	6 (30) / 8 (40) / 6 (30)
Concurrent chemotherapy	
Yes/ No	9 (45) / 11 (55)
Dose of proton radiotherapy	
median, range	50 Gy (44–56) / 25 fractions (22–28)
Dose of carbon ion radiotherapy	
median, range	21 Gy (15–23.1) / 7 fractions (5–7)

Doses are presented as the RBE (relative biological effectiveness)-weighted dose

Abbreviations: SUVmax, the maximum standard uptake value

All patients were pathologically confirmed as having SCC. All but three patients (85%) received chemotherapy. Among them, 14 (70%) patients received pre-RT chemotherapy, and 12 (60%) received post-RT chemotherapy with fluorinated pyrimidines (5-fluorouracil, capecitabine, S-1) or taxanes plus platinum. Nine (45%) received concurrent chemotherapy with taxanes plus platinum, except for one who received carboplatin monotherapy because of abnormal renal function. All patients received PRT plus sequential CIRT with a total D_RBE_ of 71 Gy (range, 65–73.1) in 32 fractions (29–33). PRT was delivered at 50 Gy (range, 44–56) in 25 fractions (22–28). CIRT was administered at 21 Gy (range, 15–23.1) in 7 fractions (5–7), 3 Gy/fraction in 7 (5–7) fractions in 13 patients and 3.3 Gy/fraction in 7 fractions in seven patients, respectively.

### Dosimetric comparison

**Fig. 1:**
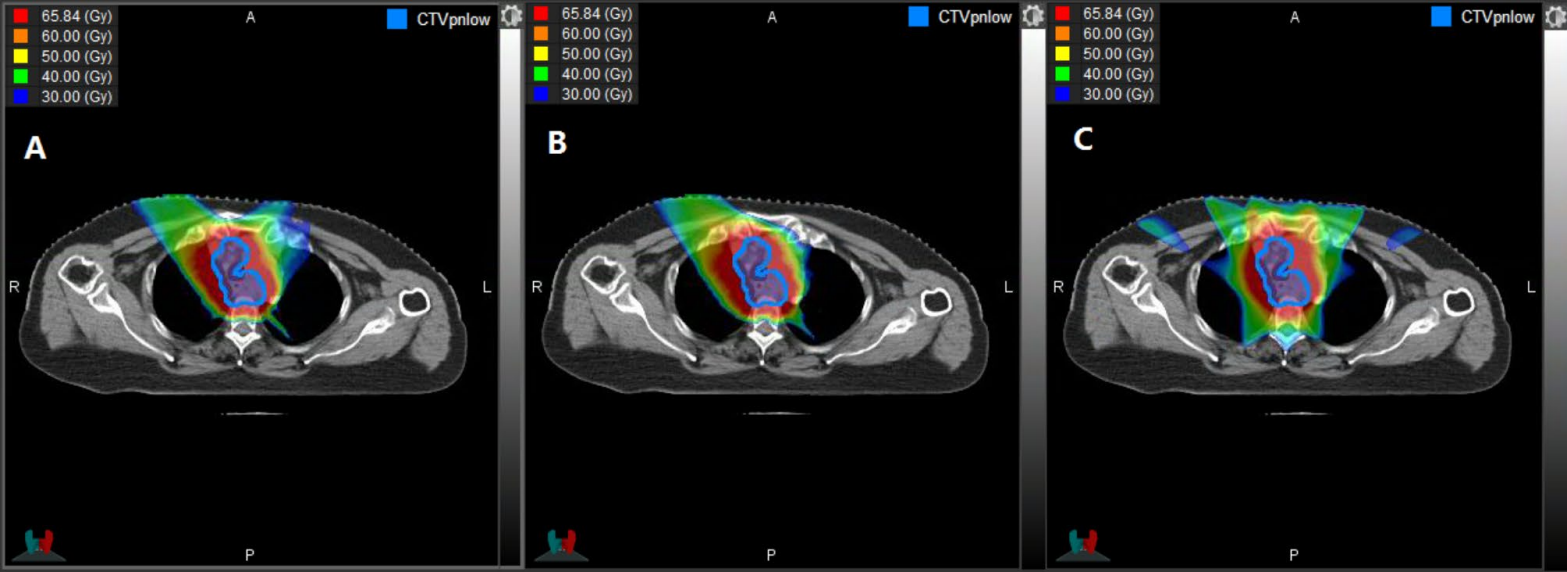
The simulated dose distribution of three radiation techniques for esophageal carcinoma. (**A**) Intensity modulated carbon-ion radiotherapy, IMCT, (**B**) Intensity modulated proton radiotherapy, IMPT, and (**C**) Photon intensity-modulated radiotherapy, IMRT. The isodose of 65.84 Gy was 95% of the prescription dose, and is presented together with levels 60, 50, 40, and 30 Gy. Doses are presented as the RBE (relative biological effectiveness)-weighted dose

The dosimetric parameters of the OARs in IMRT, IMPT, and IMCT for all patients are listed in Table 2 and illustrated in Fig. 1.

**Table 2:** Dosimetric parameters of the organs at risk in the three radiotherapy techniques studied

OARs	Characteristics	IMRT	IMPT	IMCT	*p* (IMRT vs. IMPT	*p* (IMRT vs. IMCT)	*p* (IMPT vs. IMCT)
Spinal cord	D_max_ (Gy)	48.5 ± 9.5	37.3 ± 7.4	34.3 ± 8.2	< 0.001	< 0.001	< 0.001
	D_1__%_ (Gy)	43.8 ± 9.4	30.8 ± 7.7	28.3 ± 8.8	< 0.001	< 0.001	0.001
MBT	D_max_ (Gy)	72.8 ± 1.1	71.4 ± 0.6	72.2 ± 0.9	< 0.001	0.048	0.001
	D_1__%_ (Gy)	71.4 ± 0.8	69.6 ± 0.7	70.1 ± 1.3	< 0.001	< 0.001	0.017
Heart	D_mean_ (Gy)	14.7 ± 13.3	4.9 ± 4.2	5.8 ± 5.1	< 0.001	< 0.001	0.031
	V5 (%)	44.7 ± 40.4	14.5 ± 11.3	20.2 ± 16.9	0.001	0.001	0.011
	V10 (%)	40.3 ± 38.8	11.5 ± 9.3	12.8 ± 10.7	0.002	0.002	0.031
	V20 (%)	30.1 ± 29.7	8.2 ± 7.0	8.7 ± 7.6	0.005	0.002	0.061
	V30 (%)	23.6 ± 24.4	6.3 ± 5.6	6.6 ± 5.8	0.003	0.003	0.041
	V40 (%)	12.5 ± 13.0	5.0 ± 4.7	5.1 ± 4.9	0.005	0.005	0.19
	V50 (%)	6.3 ± 6.2	4.0 ± 4.1	4.1 ± 4.3	0.005	0.005	0.40
	V60 (%)	4.5 ± 4.8	3.1 ± 3.4	3.2 ± 3.6	0.003	0.004	0.048
Lungs	D_mean_ (Gy)	12.4 ± 4.9	8.6 ± 3.4	8.9 ± 3.4	0.002	0.003	0.037
	V5 (%)	46.1 ± 18.3	31.0 ± 11.4	32.2 ± 12.1	0.001	0.002	0.093
	V10 (%)	37.3 ± 15.5	25.9 ± 9.8	25.9 ± 9.8	0.002	0.002	0.74
	V20 (%)	28.0 ± 12.5	18.2 ± 7.7	19.0 ± 7.3	0.002	0.002	0.31
	V30 (%)	15.1 ± 8.2	11.0 ± 6.1	13.3 ± 6.0	0.057	0.43	0.005
	V40 (%)	5.4 ± 3.7	5.1 ± 2.8	6.0 ± 3.1	0.91	0.17	0.017
	V50 (%)	2.8 ± 1.9	3.0 ± 1.7	3.0 ± 1.6	0.53	0.33	0.17
	V60 (%)	1.8 ± 1.2	2.0 ± 1.3	2.0 ± 1.1	0.25	0.13	0.71

**Abbreviations**: OARs, organs at risk; D_max_, maximum dose; D_1__%_, the dose that 1% of the volume received of the OARs; D_mean_, mean dose; Vx, the percentage volume of the organs at risk receiving ≥ x Gy irradiation; MBT, main bronchial tree; IMRT, intensity modulated x-ray radiotherapy; IMPT, intensity modulated proton radiotherapy; IMCT, intensity modulated carbon-ion radiotherapy. *Doses are presented as the RBE (relative biological effectiveness)-weighted dose

An adequate coverage of the tumor target was achieved in all three plans. In addition, the dose distributions to the OARs were evidently lower in the IMPT and IMCT plans than in IMRT. IMPT and IMCT showed an apparent improvement in the D_max_ to spinal cord, and in the D_mean_ to the heart and lungs compared to IMRT (p < 0.05). Of note, V5, V10, and V20 in the lungs and V5–V60 in the heart were significantly lower in IMPT and IMCT than in IMRT.

We observed a slightly higher D_max_ to the MBT and a lower D_max_ to the spinal cord in IMCT than in IMPT with statistical significance (p < 0.05). The D_mean_ and V30/V40 to the lungs and the D_mean_ and V5/V10/V30/V60 to the heart were slightly higher in IMCT than in IMPT (p < 0.05).

### Treatment toxicity

All patients completed the full PRT and CIRT course. Except in instances of unplanned failure of the treatment system, RT was interrupted in six patients, comprising myelosuppression after chemoradiotherapy in four, bacterial pneumonia in one, and esophageal ulcer in one. The patients tolerated RT and chemotherapy well (Table 3).

**Table 3:** Treatment-related toxicities of proton and carbon-ion radiotherapy combined with chemotherapy

Characteristics	Grade 1	Grade 2	Grade 3	Grade 4	Grade 5
Acute					
Pulmonary					
Cough	4 (20%)	1 (5%)	0	0	0
Pneumonitis	1 (5%)	3 (15%)	0	0	0
Gastrointestinal					
Esophagitis	12 (60%)	6 (30%)	0	0	0
General					
Weight loss	3 (15%)	0	0	0	0
Dermatitis	9 (45%)	1 (5%)	0	0	0
Hematologic					
Leucopenia	3 (15%)	2 (10%)	5 (25%)	3 (15%)	0
Neutropenia	1 (5%)	6 (30%)	0	6 (30%)	0
Anemia	14 (70%)	5 (25%)	0	0	0
Thrombocytopenia	4 (20%)	0	0	0	0
Late					
Pulmonary	10 (50%)	1 (5%)	0	0	0
Tracheal	0	0	1 (5%)	0	0
Esophageal	1 (5%)	3 (15%)	1 (5%)	0	0
Dermal	3 (15%)	0	0	0	0

Grades 3 and 4 acute hematological toxicities occurred in 10% and 30% of patients, respectively. No cases of grades 3–5 of acute non-hematological toxicity were observed. Severe late toxicities included one grade-3 pulmonary toxicity (tracheal stenosis) (5.0%) and one grade-3 esophageal toxicity (esophageal-pulmonary fistula).

### Survival and failure patterns

**Fig. 2:**
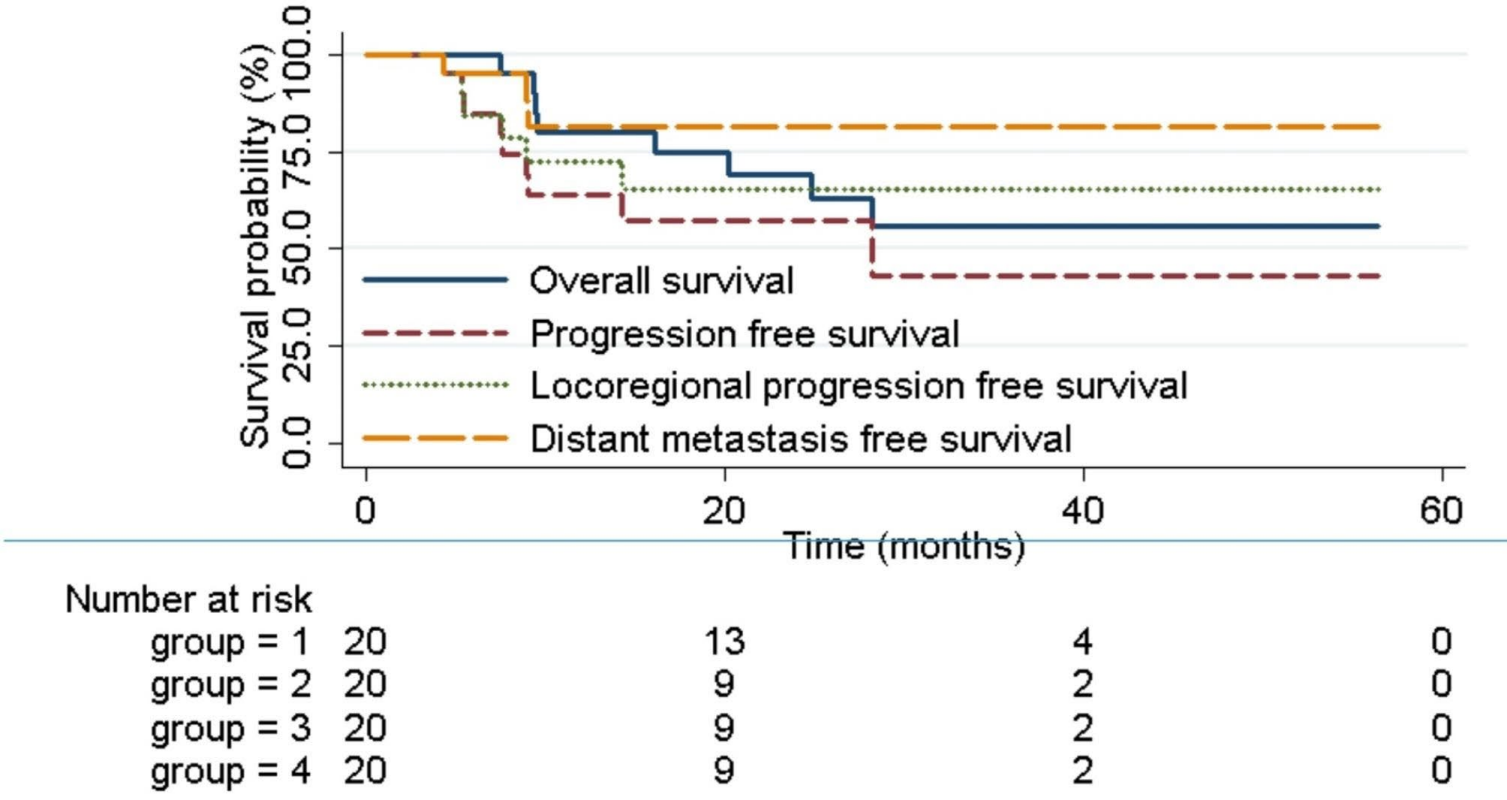
Survival probabilities of esophageal cancer patients after proton and carbon-ion radiotherapy. (Groups 1, 2, 3, and 4 delegate Overall survival, Progression free survival, Locoregional progression free survival, and Distant metastasis free survival.)

With a median follow-up period of 25.0 months (7.5–56.4), the 1- and 2-year rates of OS, PFS, LRPFS, and DMFS were 80.0% (95% CI, 55.1–92.0%), 63.8% (38.6–80.8%), 72.4% (45.6–87.6%), 81.4% (52.3–93.7%); and 69.2% (43.8–84.9%), 57.4% (32.2–76.2%), 65.1% (37.6–82.9%), 81.4% (52.3–93.7%), respectively (Fig. 2). The median times of OS, LRPFS, and DMFS were not reached. The median PFS time was 28.2 months.

At the last follow-up, 12 patients (60.0%) were alive, with a median follow-up period of 29.2 months (16.3–56.4). Among these patients, seven experienced recurrence, comprising four with locoregional failure, one with distant failure, and two with both locoregional and distant failure.

## Discussion

In the current study, we delivered PRT plus CIRT with PBS to 20 patients with primary esophageal SCC. With a median follow-up period of 25.0 months, 2-year OS and PFS rates of 69.2% and 57.4%, respectively, were achieved with mild RT-related side effects in a cohort of patients with stage II–III and IV (limited to patients with metastasis only to paraesophageal supraclavicular and cervical non-regional lymph-node) and a median age of 70 years. PRT and CIRT produced lower doses to the OARs than did photon IMRT, notably as D_max_ to the spinal cord and D_mean_ to the lungs and heart. Dosimetric comparison indicated a rationale for PRT and CIRT in esophageal carcinoma. PRT plus CIRT with PBS appears safe and effective for esophageal carcinoma in a short-term observation.

Concurrent chemoradiotherapy was recommended for patients with locally advanced esophageal carcinoma who were not candidates for resection or could not tolerate surgery, in keeping with its preferred status in such patients [26]. Photon radiotherapy concurrently with chemotherapy has shown efficiency and tolerability compared to photon radiotherapy alone against locally advanced esophageal carcinoma, with 2- and 3-year OS rates of 36% and 30% after combined chemoradiotherapy, compared to 10% and 0% in the radiotherapy-only group, respectively [27]. An RT dose of 50–50.4 Gy was demonstrated to be highly acceptable for patients with locally advanced esophageal carcinoma [5]. The 2-year OS rates were typically 36–55% in randomized studies and the highest, reported recently, was 67% [6, 27–29].

In trials of neoadjuvant chemoradiotherapy, [30–32] a complete response rate of 30–40% after 40–50 Gy was noted in esophageal carcinoma, indicating that an irradiation dose of 40–50 Gy is sufficient for one-third of esophageal carcinoma patients. However, for the remainder, a higher dose might be indicated if the attendant toxicity was tolerable. A population-based, propensity-score–matched analysis suggested that a higher irradiation dose (≥ 60 Gy vs. 50–50.4 Gy) might improve survival in patients with esophageal SCC [33]. However, a randomized study showed that a total dose of 64.8 Gy did not improve clinical outcomes compared with 50.4 Gy in locally advanced esophageal carcinoma and showed an extended treatment duration due to toxicity breaks and a decreased actual dose of fluorouracil [6]. Furthermore, a recent randomized study (ARTDECO) reported similar OS and LPFS rates between RT doses of 50.4 and 61.6 Gy [28]. Analysis of patients with esophageal carcinoma receiving PRT or IMRT showed that PRT and a D_mean_ of < 15 Gy to the heart are associated with a decreased incidence of severe (grade 3 or higher) cardiac events, and these events are associated with poorer OS (p < 0.05) [34]. These findings indicate that technical improvements in highly conformal therapy can be expected to improve clinical results in locally advanced esophageal carcinoma if they lead to an increased dose to targets without increased radiation exposure to the OARs (especially the heart).

Similar to our results, the literature also shows that protons and carbon ions can better protect normal tissues than can photons, specifically 3DCRT or IMRT, whether they are passive scattering protons or PBS beams. Compared to IMRT, PRT achieved a significant decrease with the passive scattering technique, especially a decline in the D_max_ of the spinal cord, D_mean_, V25, V30, V40, and V50 of the heart and D_mean_, V5, V10, and V15 of the lungs (Table 4) [9]. CIRT with PBS (i.e. IMCT technique, similar to the technique used in our center) produced better dose homogeneity in the target volume and significantly lower doses to the heart, lungs, spinal cord, and skin in esophageal carcinoma than did photon 3DCRT or volumetric modulated arc therapy (VMAT, a variant of IMRT). Compared to VMAT, significant decreases were achieved with CIRT, especially in the D_max_ to the spinal cord; D_mean_, V10, V20, V30, and V40 to the heart; and D_mean_, V5, V10, V20, V30, and V40 to the lungs [35].

**Table 4:** Dosimetric parameters of organs at risk in esophageal carcinoma treated with photon vs. particle radiotherapy

OARs	Characteristics	Ling et al. (50.4 Gy) [9]					Takakusagi et al. (50.4 Gy) [35]				
		Proton	IMRT	p	3DCRT	p	Carbon	VMAT	p	3DCRT	p
Spinal cord	D_max_ (Gy)	11.6 ± 10.0	36.9 ± 3.5	0.001	31.2 ± 9.7	0.001	25.6 ± 3.5	41.3 ± 2.9	< 0.001	44.6 ± 0.6	< 0.001
Lungs	D_mean_ (Gy)	6.0 ± 2.6	9.5 ± 3.2	0.016	9.4 ± 4.0	0.040	1.8 ± 0.9	11.4 ± 2.3	< 0.001	5.3 ± 1.8	< 0.001
	V5 (%)	21.4 ± 10.3	46.9 ± 17.6	0.001	34.1 ± 13.9	0.032	8.4 ± 3.7	77.7 ± 17.8	< 0.001	21.6 ± 8.3	< 0.001
	V10 (%)	19.4 ± 8.6	37.8 ± 14.7	0.003	29.1 ± 12.7	0.060	6.7 ± 3.2	52.5 ± 12.1	< 0.001	14.8 ± 5.5	< 0.001
	V20 (%)	15.3 ± 6.5	16.2 ± 5.8	0.794	22.1 ± 10.8	0.114	2.6 ± 1.7	14.8 ± 5.5	< 0.001	7.7 ± 3.3	< 0.001
	V30 (%)	6.1 ± 2.9	6.6 ± 3.2	0.720	9.8 ± 5.1	0.067	1.4 ± 1.0	3.9 ± 2.3	< 0.001	5.5 ± 2.5	< 0.001
	V40 (%)	4.3 ± 2.1	3.5 ± 2.0	0.391	4.7 ± 2.9	0.682	0.3 ± 0.5	1.0 ± 0.9	0.001	2.2 ± 1.2	< 0.001
	V50 (%)	1.1 ± 1.0	1.6 ± 1.3	0.251	3.3 ± 2.1	0.008	0.0 ± 0.1	0.0 ± 0.1	0.157	0.1 ± 0.2	0.006
Heart	D_mean_ (Gy)	12.6 ± 3.9	28.5 ± 5.5	0.001	27.5 ± 5.2	0.001	9.6 ± 4.5	22.3 ± 9.0	< 0.001	29.1 ± 11.7	< 0.001
	V10 (%)	N/A	N/A	N/A	N/A	N/A	46.5 ± 21.0	99.6 ± 39.6	< 0.001	69.2 ± 28.1	< 0.001
	V20 (%)	N/A	N/A	N/A	N/A	N/A	9.8 ± 5.6	45.0 ± 24.1	< 0.001	61.4 ± 25.8	< 0.001
	V30 (%)	20.9 ± 7.1	42.3 ± 15.3	0.001	32.7 ± 9.4	0.005	6.7 ± 3.7	18.0 ± 9.6	< 0.001	57.3 ± 24.8	< 0.001
	V40 (%)	16.2 ± 6.4	25.5 ± 11.0	0.036	25.8 ± 8.8	0.012	4.8 ± 2.6	7.3 ± 5.0	< 0.001	51.1 ± 23.5	0.001
	V50 (%)	2.8 ± 2.3	12.0 ± 8.6	0.008	20.0 ± 12.6	0.002	0.9 ± 0.6	1.1 ± 1.0	0.068	8.3 ± 6.6	0.006

Doses are presented as the RBE (relative biological effectiveness)-weighted dose

**Abbreviations**: OARs, organs at risk; D_max_, maximum dose; D_mean_, mean dose; IMRT, intensity-modulated radiotherapy; 3DCRT, 3D conformal radiotherapy; VMAT, volumetric modulated arc therapy; Vx, the percentage volume of the organs at risk receiving ≥ x Gy of irradiation; N/A, not available

To our knowledge, this is the first study to compare dosimetric parameters among IMPT, IMCT, and photon IMRT in esophageal carcinoma. Similar to Ling et al.’s study using the scattering technique, [9] in our study, PRT using the scanning technique produced a more apparent decline in V20 of the lungs than did IMRT. Compared with PRT using passive scattering, [10] the more advanced IMPT with PBS is associated with further dosimetric benefits in sparing the heart in radiotherapy for esophageal cancer and has also been reported as sparing the lungs and heart in radiotherapy for lung cancer [36]. CIRT using the scanning technique here produced results for esophageal cancer similar to those reported by Takakusagi et al., [35] which included a similar decline in the D_max_ of the spinal cord, a decrease in the D_mean_ and V5–V60 of the heart, and a lesser decline in the D_mean_ and V5–V20 of the lungs. Those authors used the same PTV for photon and carbon-ion planning; in contrast, in this study, a larger PTV (beam-specific PTV) was applied as in routine clinical practice of particle therapy. Although a trend of better dose distribution to OARs was noted in PRT compared to CIRT planning, the differences were minor in this study.

By taking advantage of dose distribution, PRT led to either a lesser treatment-related toxicity burden than photon radiotherapy or an improvement in survivals for esophageal cancer [10, 11]. Xi et al. conducted a retrospective study of PRT and photon IMRT with a total dose of 50.4 Gy/28Fx concurrently with chemotherapy of fluorouracil and platinum/taxane in esophageal carcinoma [10]. PRT led to improved survival, with higher 5-year OS and PFS rates of 41.6% and 34.9% compared with photon radiotherapy rates of 31.6% and 20.4%, respectively. PRT also induced low severe toxicities compared with photon radiotherapy, with treatment-related severe toxicity of 39% vs. 47% (p > 0.05). Furthermore, a prospective phase-IIb randomized study of PRT mostly using a passive scattering technique vs. photon IMRT with a total dose of 50.4 Gy/28Fx concurrently with fluorouracil/taxane-based chemotherapy showed that PRT maintained similar OS and PFS rates (3-year OS, 51.2% vs. 50.8%; 3-year PFS, 44.5% vs. 44.5%). However, PRT was associated with a lower total toxicity burden [11]. Failure to improve clinical outcomes may be related to the passive scattering technique initially used and a lack of clinician experience with the new technology, similar to the lesson learnt with lung cancer. Compared to PRT, our study on IMPT followed by IMCT with a median total dose of 71 Gy (range: 65–73.1) in esophageal carcinoma produced a promising clinical outcome (2-year OS, 69.2% and 2-year PFS, 57.4%) with mild esophageal toxicities (5%). Severe esophageal and pulmonary toxicity rates of more than 20% were observed after photon RT and PRT with passive scattering in early patients, which included a severe late esophageal toxicity rate of 19–21% after photon RT and severe esophagitis and esophageal stricture rates of 11.4% and 9.8% after proton RT, respectively [6, 10, 27]. The clinical benefit of this treatment modality may be attributed to the dosimetric advantages of IMPT and IMCT when combined with PBS, which can further reduce the dose to normal tissue compared with passive scattering technique, thereby further reducing doses to the heart and thus the incidence of adverse events.

This study had some limitations. First, a definitive conclusion could not be drawn owing to the small sample size and the retrospective nature of the study. Second, our study delivered a wide range of dose prescriptions, attempting to balance toxicity and clinical efficacy. Finally, this study included patients with stage IV esophageal cancer (with metastasis only to paraesophageal non-regional lymph nodes, defined in AJCC, 8th edition), which hampered a definitive conclusion for locally advanced esophageal cancer. Currently, a large-scale prospective study to evaluate the efficacy and safety of PRT plus CIRT in locally advanced esophageal carcinoma is underway. We expect that the results will validate our findings.

## Conclusions

This is the first study to investigate combined IMPT and IMCT for esophageal carcinoma. Our results demonstrated better sparing of OARs with mild toxicities compared to IMRT and showed promising OS and PFS rates. Further prospective studies with more patients are warranted.

## Data Availability

The datasets generated and/or analyzed during the current study are not publicly available due to hospital policy but are available from the corresponding author on reasonable request.

## Abbreviations

3DCRT: 3D conformal radiotherapy
CI: Confidence interval
CIRT: Carbon-ion radiotherapy
CT: Computed tomography
CTCAE: Common terminology criteria for adverse events
CTV: Clinical target volume
D_1%_: The dose that 1% of the volume received
D_max_: Maximum dose
D_mean_: Mean dose
DMFS: Distant metastasis-free survival
D_RBE_: RBE-weighted dose
DVH: Dose volume histogram
GTV: Gross tumor volume
iGTV: Internal gross tumor volume
IMCT: Intensity modulated CIRT
IMPT: Intensity modulated PRT
IMRT: Intensity-modulated radiotherapy
LEM: Local effective model
LET: Linear energy transfer
LRPFS: Locoregional progression-free survival
MBT: Main bronchial tree
OAR: Organ at risk
OS: Overall survival
PBS: Pencil beam scanning
PET: Positron emission tomography
PFS: Progression-free survival
PRT: Proton radiotherapy
PTV: Planned target volume
RBE: Relative biological effectiveness
RT: Radiotherapy
SCC: Squamous-cell carcinoma
SUV_max_: Maximum standard uptake value
VMAT: Volumetric modulated arc therapy
Vx: Percentage volume of the lungs receiving ≥ x Gy of irradiation
